# GENDER, HEALTH, AND EXTENDED WORKING LIFE IN IRELAND

**DOI:** 10.1093/geroni/igz038.3023

**Published:** 2019-11-08

**Authors:** Aine Ni Leime

**Affiliations:** National University of Ireland, Galway Ireland, Galway, Ireland, Ireland

## Abstract

This presentation is based on a forthcoming book chapter which analyses policies, statistical evidence and qualitative data to investigate the gender and health implications of Extended Working Life policy in Ireland. The qualitative data is from a study conducted in 2018 that investigated attitudes to extended working life and experiences of late life work among sixty older workers, 30 men and 30 women in three different occupations, health care workers/cleaners, teachers and academic faculty.The data were analysed using a lifecourse approach. Workers in physically-demanding occupations, those in precarious employment and women were found to be more likely to be disadvantaged in relation to options for extending their working lives. It is recommended that policies be modified to address the disadvantages faced by these groups of workers.

